# VoiceLove for patient-family communication in the ICU: a usability study of a novel voice messaging app

**DOI:** 10.3389/fmed.2026.1838229

**Published:** 2026-10-07

**Authors:** Soibhan Kelley, Leanne M. Boehm, Mariu Duggan, Jason M. Slagle, Carrie Reale, Russ Beebe, E. Wesley Ely, Christina Boncyk

**Affiliations:** 1 Division of Allergy, Pulmonary, and Critical Care Medicine, Department of Medicine, Vanderbilt University Medical Center (VUMC), Nashville, TN, United States; 2 Critical Illness, Brain Dysfunction, and Survivorship (CIBS) Center, VUMC, Nashville, TN, United States; 3 Vanderbilt University School of Nursing, Nashville, TN, United States; 4 Division of Geriatric Medicine, Department of Medicine, VUMC, Nashville, TN, United States; 5 Division of Critical Care Medicine, Department of Anesthesiology, VUMC, Nashville, TN, United States; 6 Center for Research and Innovation in Systems Safety, VUMC, Nashville, TN, United States; 7 VA Geriatric Research Education Clinical Center (GRECC), Tennessee Valley Veterans Affairs, Nashville, TN, United States

**Keywords:** mobile applications, user-centered design, communication barriers, intensive care unit, family, communication

## Abstract

**Background:**

Family engagement has been associated with improved outcomes following critical illness, but communication barriers challenge family engagement. Traditional video or phone call strategies to navigate communication barriers are time-, resource-, and labor-intensive for hospital staff, often limiting their use. VoiceLove is a Health Insurance Portability and Accountability Act (HIPAA)-compliant mobile phone app that enables family members to send real-time voice messages to intensive care unit (ICU) patients without relying on the clinical team to coordinate calls. We conducted iterative usability testing of VoiceLove to optimize use for patients, families, and their clinical teams in the adult ICU.

**Methods:**

We completed 2 rounds of usability testing of the VoiceLove app in a simulated environment. Usability was assessed through System Usability Scale (SUS) scores and task success rates. Semi-structured interviews were used to identify themes, concerns, and design priorities.

**Results:**

Mean SUS scores for rounds 1 and 2 of testing were 77.5 (SD 11.1) and 75.9 (SD 26.8), respectively. The app concept was overall viewed favorably as a tool with the potential to provide comfort and streamline communication. Key usability issues included navigation, clarity of labels, and accessibility, which were addressed through iterative app improvements. Implementation concerns included nursing workload, family cognitive burden, and interplay with complex family dynamics.

**Conclusion:**

A diverse group of ICU stakeholders found the VoiceLove app demonstratedpotential to improve communication with critically ill patients inherently disadvantaged in their ability to communicate. Further testing in a real-world setting is needed to assess the impact of VoiceLove implementation on clinical team work load and family dynamics

## 1 Introduction

There is increasing recognition of the importance of delivering both patient- and family-centered care in the intensive care unit (ICU) on both patient and family outcomes following hospitalization. Liberalized ICU visitation policies have been associated with reduced symptoms of anxiety, depression, and post-traumatic stress disorder (PTSD) for patients and family members and to improve family satisfaction with care (1–3). Qualitative initiatives designed by ICU teams to enhance family engagement, including family-centered councils and family presence during multidisciplinary rounds, have also been associated with improved perceptions of care quality and satisfaction with care (4, 5).

Family presence in the ICU may also be protective against delirium, a common and devastating disturbance in cognition and associated with higher mortality (6–8), longer duration of mechanical ventilation (8), greater ICU and hospital days (7, 8), and long-term cognitive impairment (6, 8–10). Family engagement is a key pillar in the ABCDEF Bundle designed to promote ICU liberation with a specific focus on delirium management (11). Even remote family presence may have therapeutic value. Family visitation, whether in-person or virtually, has been associated with decreased prevalence of delirium in critically ill patients with COVID-19 (12) and decreased duration of delirium in medical and surgical ICU patients (13). Similarly, mechanically ventilated patients who were played pre-recorded audio messages in a family member's voice, with orientating and reassuring content, experienced more delirium-free days than those who received usual care (14).

While keeping patients and families connected during critical illness is paramount to providing humanistic care and improving outcomes, there are barriers to optimizing family engagement. Family members cannot always be at the bedside due to time constraints, competing obligations (e.g., childcare and job-related duties), geographic separation, and transportation limitations. ICU patients also struggle to facilitate communication due to cognitive impairment, restraint use, intubation, presence of medical devices, and limited dexterity. Facilitating patient-family communication through traditional means, such as video or phone calls, often requires substantial time, staff effort, and resources in the ICU. Digital approaches to family engagement are therefore increasingly relevant. In an integrative review of adult ICU interventions, digital tools such as web-, tablet-, SMS-, and video-based platforms supported information sharing, family participation, and access to conferences or rounds (15).

VoiceLove is a novel, Health Insurance Portability and Accountability Act (HIPAA)-compliant mobile app created in response to the communication crisis of the COVID-19 pandemic. Like a walkie-talkie, it enables family members to send voice messages from their own phone that are played in real-time for ICU patients via a bedside-mounted phone, without requiring continuous staff coordination. The app also allows a healthcare proxy to create separate groups for family or friends, which can streamline communication and tailor sharing across audiences.

While VoiceLove shows promise for supporting family engagement in the ICU, it has not yet been rigorously evaluated to ensure that it meets the needs of ICU patients, families, and clinical teams. In this study, we aimed to identify ICU stakeholder priorities for design and function of a voice-messaging based communication tool, evaluate the usability of the VoiceLove app, and identify potential barriers to VoiceLove implementation in the ICU. We conducted iterative usability testing of VoiceLove in a simulated setting with diverse ICU stakeholders, using quantitative performance measures and qualitative feedback, to identify usability and implementation concerns. These findings informed evidence-based recommendations to refine and optimize the app to improve usage among end users before a future interventional trial.

## 2 Methods

An overview of study processes is shown in Figure 1. End-users for usability testing included a convenience sample of participants identified within participating ICUs following local institutional review board (IRB) approval. Family members were included if they had exposure to the ICU, were proficient in English or Spanish, and agreed to participate. Clinician stakeholders were included if they had work exposure within the ICU and agreed to participate. We excluded non-English or Spanish speakers and any family members or staff who to declined to participate. For the formative usability evaluations, we sought a minimum sample size of 4–5 representative participants in each round of iterative testing, consistent with prior work showing that this sample size can identify approximately 80% of usability problems and achieve a pragmatic balance between resource constraints and problem discovery (16).

**Figure 1:**
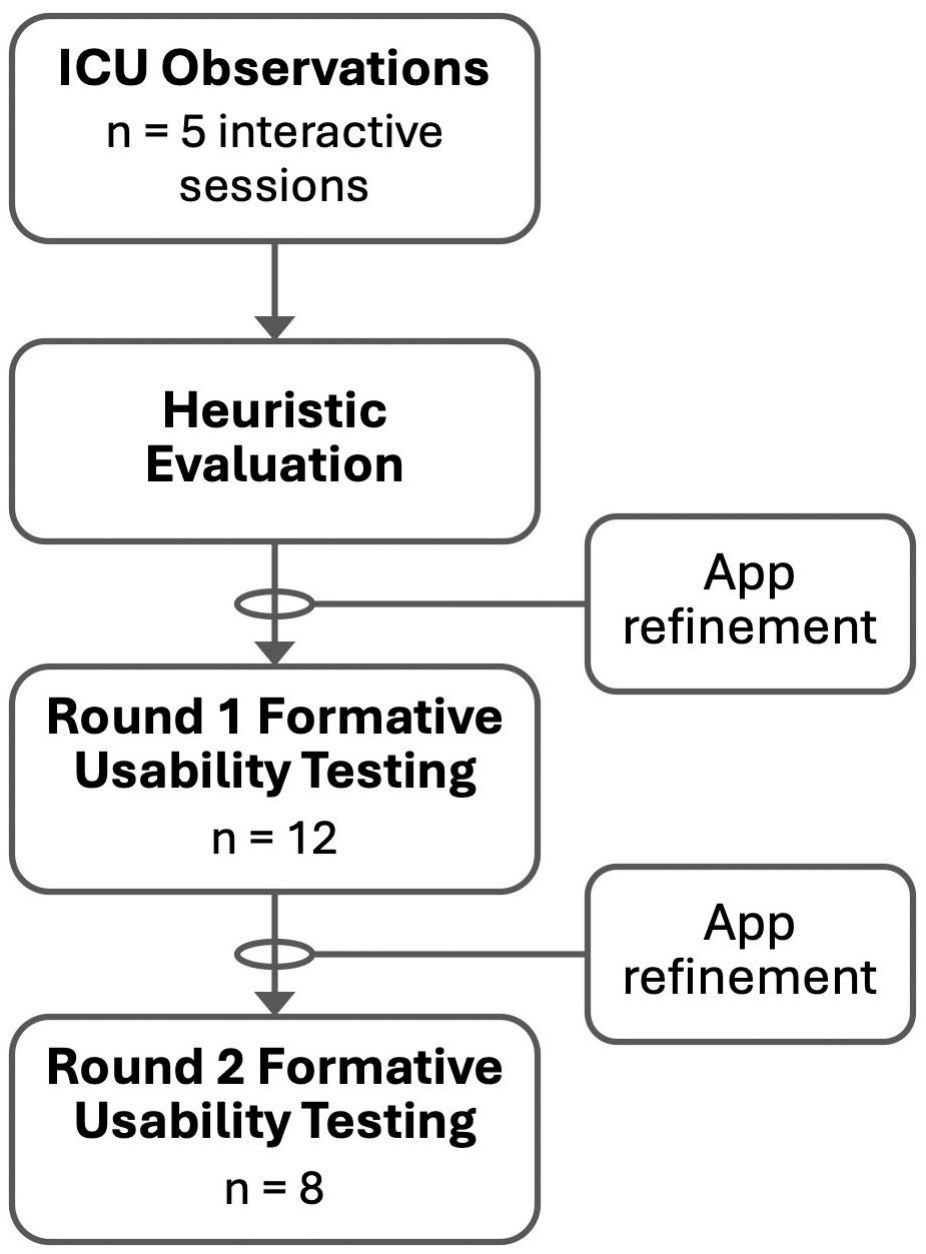
Descriptive design of study progression through ICU observations to understand existing patient-family communication processes, heuristic evaluation of the VoiceLove app with human factors engineers, and usability testing using a live prototype with a diverse range of end users. Round 1 of usability testing focused on clinical staff including nurses and physicians. Round 2 focused on family members of ICU patients with additional input from 2 nurses. Iterative app refinements were made throughout the process as shown above.

### 2.1 On-site observations and heuristic evaluation

Prior to end-user testing, our team, comprised of subject matter experts in user-centered design (UCD) and human factors research from Vanderbilt University's Center for Research and Innovation in Systems Safety (CRISS), conducted interactive observations in the medical ICU to understand the current workflow for communication between ICU patients and their families and to identify potential implementation barriers. The observations were conducted over 5, 2-hour long interactive sessions that included input from nurses, medical receptionists, and physicians.

After completing unit observations, three human factors engineers (CR, RB, JS) completed a heuristic evaluation of the VoiceLove app to identify usability problems and suggest design modifications based on design standards and industry best practices (17). Feedback was used to make app adjustments ahead of usability testing.

### 2.2 Formative usability testing

After completion of the heuristic evaluation and subsequent app revisions, we proceeded with iterative usability testing targeting a variety of end users with local IRB exemption (#230545). Usability testing included 2 rounds of data collection, the first of which focused on a variety of ICU team members while the second included additional ICU team members and incorporated family participants. The first session (*n* = 12) included 6 nurses (2 each from medical ICU, cardiovascular ICU, and an inpatient geriatric unit), 1 care partner, 1 chaplain, 1 geriatrician, and 3 ICU fellow physicians. The second session (*n* = 8), conducted 7 months later with a refined prototype, included 6 family members of ICU patients who were recruited from our medical and cardiovascular ICUs and 2 ICU nurses. Participant demographics are detailed in Table 1.

**Table 1:** Demographics of usability testing participants. Round 1 included clinical team members. Round 2 included primarily family members of ICU patients in addition to 2 nurses.

Characteristic	All participants *n* = 20	Round 1 testing *n* = 12	Round 2 testing *n* = 8
Female; *n* (%)	16 (80)	9 (75)	7 (88)
Age; *n* (%)			
18–29	6 (30)	4 (33)	2 (25)
30–39	7 (35)	6 (50)	1 (13)
40–49	2 (10)	1 (8)	1 (13)
50–59	2 (10)	1 (8)	1 (13)
60+	3 (15)	0 (0)	3 (37)
Highest education level, *n* (%)			
High school	1 (5)	0 (0)	1 (13)
Some college	4 (20)	0 (0)	4 (50)
College degree	10 (50)	7 (58)	3 (37)
Graduate degree	5 (25)	5 (42)	0 (0)
Comfort with technology*; *n* (%)			
Early adopter	1 (5)	1 (8)	0 (0)
Early majority	14 (70)	11 (92)	3 (37)
Late majority	5 (25)	0 (0)	5 (63)

* Self-identified comfort level with technology. Early adopters are the first among their peers to adopt new technology, early majority users like to see how new technology works for others before adopting it, and late majority users tend to wait until technology is well established before adopting it.

An informatics research nurse (CR) with expertise in human factors engineering and usability testing facilitated all sessions. Sessions were an hour long and included audio recording with transcription and video recording of device interactions. Participants completed a set of standardized tasks focused on basic app functions on a live Android test device. For the family members, tasks included activating an account within the app, adding a channel (a customizable group message), inviting other family members or friends to the channel, favoriting a message, and sending a message to a patient. Tasks for clinician users included assigning a device to a patient, requesting messages from the healthcare proxy, playing a message to a patient, placing device in a prototype of a soft plastic mounting case (Figure 2), recording a message, and unassigning a device. Participants also reviewed screenshots of other app design concepts.

**Figure 2:**
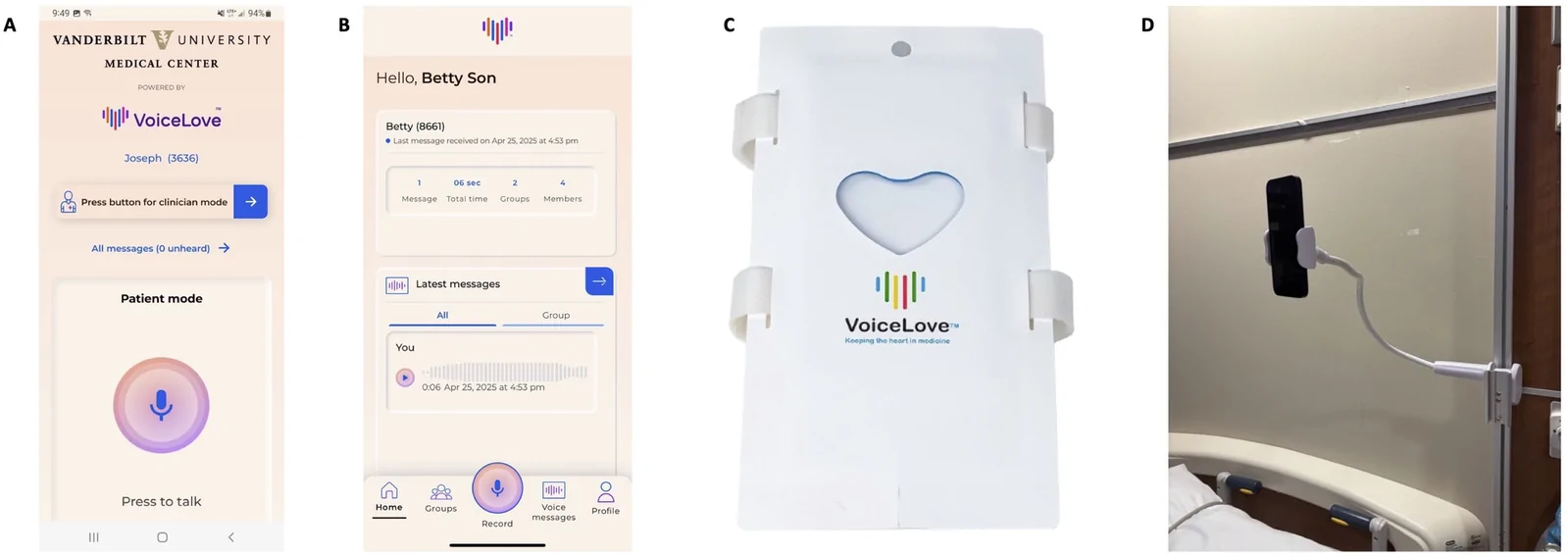
Selected screenshots of app prototype and pictures of mounting devices. **(A)** Panel A* shows the patient facing VoiceLove prototype home screen. This screen would be visible on the phone that is mounted at the ICU bedside. Voice messages play automatically for patients in real time. A large, easily accessible record button can be used by the patient to record messages for their family members if desired. Clinical team members can quickly access menu options from this screen to use features such as “do-not-disturb.” **(B)** Panel B* shows the family facing VoiceLove prototype home screen. This screen allows the health care proxy and other approved family members to send messages to the patient and to family/friend groups from their personal phone. Messages can also be replayed and favorited. **(C)** Panel C shows the original design of the case used to store the phone at the patient bedside. The phone was inserted into this soft plastic sleeve that was designed to hang from a hole at the top of the case by the bedside (e.g., on IV Poles). **(D)** Panel D shows the revised mounting system incorporating feedback from usability testing and bedside ICU nurses. The mount is a hard plastic clamp that attaches to the existing medical equipment rail located near the head of the ICU bed. This system keeps the phone secure and within earshot but is unobtrusive to clinical tasks. *Names used in the app prototype screenshots represent fictional testing subjects.

We analyzed performance metrics for each session including task completion rates, use errors, and task completion times. All errors were weighted the same. After task completion, participants completed the System Usability Scale (SUS), a validated 10-item questionnaire used to measure the perceived usability of applications (18). A standardized SUS score was then calculated with scores ranging from 0 (worst) to 100 (best). At the end of each testing session, semi-structured debrief interviews were conducted to solicit more open-ended feedback related to their experiences using the app and their related opinions and suggestions. These session debrief transcripts were independently reviewed by the same informatics research nurse (CR) and an additional study team member (SK). Transcripts were reviewed to extract additional context for the usability issues discovered during task performance. This allowed us to identify additional relevant themes pertaining to implementation concerns, identify specific functions and aspects of the app that caused confusion, delays, inability to complete tasks, or errors, and to elicit suggestions to resolve such issues as well as to prioritize stakeholder needs to improve the design, functionality, and usability of the app.

### 2.3 Statistical analysis

We used descriptive statistics to describe user characteristics. The overall SUS score was calculated as the sum of the total of each corrected item (subtract 1 from odd number items, subtract even number items from 5 to create a 4-point scale) multiplied by a 2.5 conversion factor to create the highest score of 100.

We used descriptive statistics to present means (standard deviation), medians (interquartile range), and frequency (proportion) to describe key participant feedback and participation metrics such as enrollment rate, attrition, survey completion, and task completion.

## 3 Results

### 3.1 On-site observations and heuristic evaluation

ICU observations identified that existing workflow to coordinate communication between patients and their family members is cumbersome and typically involves multiple phone calls and coordination among several staff members. Communication initiated by a family member is approved through a patient-specific security code after calling into the ICU. The call is subsequentially triaged by the medical receptionist to the beside ICU nurse, who will provide updates at the time of the call, if able to do so at that time, or will ask the family member to call back later, if needed, due to clinical obligations. Nurses are often asked to relay messages to the patient from family members, even for patients that are awake and have access to a personal phone. These messages are relayed to the patient when time allows in the nurse's workflow.

During ICU observations, nurses generally viewed the concept of using the VoiceLove app favorably as a tool to facilitate communication between patients and family members. Several nurses noted significant emotional burden and increased cognitive workload to accurately relay detailed and deeply personal messaged to their patients. Nurses also noted that the app could help children communicate with ICU patients, as children may feel too overwhelmed or scared to speak by phone in real time but may find it easier to record and send messages in a more controlled, unhurried setting.

“It [VoiceLove app] would give me satisfaction to better support my patients with this need.”

*-* ICU nurse during on-site observations

“With this tool, family could have confidence that their messages are delivered unaltered in their own voice”

*-* ICU nurse during on-site observations

We explored the concept of using the VoiceLove app to communicate between family members and the care team as well. Nurses expressed some interest, but also reported concerns about patient privacy, increased workload, and accuracy of recorded information in the ICU where clinical status can change rapidly. Physicians generally did not support using the VoiceLove app for medical updates, citing privacy and medicolegal concerns, limitations of one-way communication for complex information, and risk of added workload if messages prompted more calls to nurses. Physicians preferred using VoiceLove as a tool to promote family engagement and patient-family communication.

Implementation concerns included the need to develop clear processes for managing the equipment. Considerations included where to securely store phones and how to track them to prevent loss and theft, charging, positioning in the room to be audible but not disruptive to clinical tasks, and cleaning protocols.

During heuristic evaluation, key usability issues included navigation among different screens, the message recording process, and managing received messages. The heuristic evaluation findings resulted in recommendations on specific app modifications and protypes to streamline and standardize screen navigation, improve messaging accessibility by eliminating the need to hold a button to record, and to optimize the message management screen. It was also suggested to add a “do-not-disturb” feature to silence messages for clinical care or sleep.

### 3.2 Formative usability testing

#### 3.2.1 Satisfaction scores

Quantitative measures of app usability suggested that users overall were satisfied with app usability. The mean SUS score in round 1 was 77.5 (SD: 11.1) with a range of 57.5 to 95. The median SUS score was 80. The mean in round 2 was 75.9 (SD: 26.8) with a range of 17.5 to 100 noting that the lowest score of 17.5 was an outlier from one family user with the next lowest score being 60. Median round 2 SUS score was 82.5. Based on industry research, a mean SUS score of 68 suggests “above average” usability (19).

#### 3.2.2 Task performance

During round 1 testing with clinical staff, overall task success rate was high at 90% with 6 of 8 task failures related to placing the patient-facing phone into “case mode”. This mode was used when the phone was placed in a proprietary soft plastic case for bedside use. Several task failures were related to difficulty placing the phone in the case, which users found confusing. Based on usability testing and nurse feedback, the soft plastic case was replaced. This feedback informed the design of a case that was easier to use and clean and better suited for integration at the ICU bedside without disrupting clinical care. Images of the original and revised mounting systems are shown in Figure 2.

During round 2, clinician task success rate was improved and very high (i.e., 98%). Family member task success rate was 76% with errors evenly distributed amongst creating groups, sending messages to specific groups or people, and message management including “favoriting” and “forward” task types.

#### 3.2.3 Post-task debrief interviews

Perceptions of the app concept were generally positive. Both clinicians and family members reported that the ability to send direct voice messages could be useful for ICU patients and their families. Users valued that messages played automatically for patients without staff coordination for communication and felt that hearing familiar voices could comfort patients. Family members also valued the app's potential to share patient status updates within the family, which could reduce the burden of the healthcare proxy making multiple phone calls.

“Yeah, it's sometimes just hearing voices and people care about you. It's better than a text message because, you know, we've got that voice there. You're that familiar person.”

*-* Family member of an ICU patient

“I have family that's out of town and it would be a nice way to update everybody without having to text this person, text that person, text this person, text that person.”

*-* Family member of an ICU patient

Table 2 summarizes usability themes and implementation concerns with suggested changes or solutions. Usability themes included: (1) navigation, (2) clarity of labels, and (3) accessibility. Implementation concerns included nursing workload, cognitive load on family members, and interface with complex family dynamics.

**Table 2:** Summary of key usability and implementation concerns with solutions.

Usability issues	
Issue description	Solutions
Navigation Menu locations were inconsistent on different screens Tasks required frequent repeated icon clicks Lack of obvious home screen to reorient users	Home screen created with clearer menu options Basics tasks within the app were streamlined to decrease amount of required clicks Menu options and headings were standardized across different screens
Confusing labels Icons with pictures only were confusing, particularly for older users Messaging groups were labeled as “channels” and users were unsure what this function was	Words were added to pictures on all icons The term “channels” was changed to “groups”
Accessibility Font was too small throughout the app Icons were small and close to screen edge	Font sizes increased where able Icons were moved and made larger
Implementation concerns	
Concern	Proposed solutions
Nursing workload App could add to nursing workload if patients and family members need frequent help with troubleshooting	Provide resources for app education and troubleshooting to family members and patients Resources could include handouts, instructional videos, in-app frequently asked questions
Burden to family members Concern that learning a new app could place burden on family members, particularly older users or those less familiar with technology “*For people who are in a fog, they just don't need one more thing to have to try to, you know, figure out.”—*Family member	Provide resources for app education as detailed above Keep app design and purpose simple
Potential to amplify complex family dynamics VoiceLove app is a new avenue where family conflicts could unfold Messages with family conflict could be disruptive for patients and family members	Healthcare proxy has full control over the app and who is invited into a group Add the ability to flag concerning messages so the proxy can quickly identify and remove concerning content or members

Family members had several ideas for new app features to be considered. This included the ability to share photos and videos, flag messages as favorites or priority, save messages after discharge as keepsakes, particularly if a patient died, and support multiple languages.

## 4 Discussion

Current methods for communication between ICU patients and family members are cumbersome and rely heavily on clinical staff to coordinate calls. In our observations, family messages often had to be relayed through multiple phone calls and ultimately depended on nursing time for delivery. The VoiceLove app emerged as a novel technology with the potential to connect patients and their loved ones more directly, without relying on day-to-day coordination from the clinical team. These findings add to a growing literature suggesting that digital tools can support family engagement in the ICU, particularly when they reduce practical barriers to connection and create more consistent opportunities for participation.

In our usability testing, diverse ICU stakeholders including clinical team members and families viewed the concept of VoiceLovefavorably. Highlighted strengths included the potential for voice messages to comfort patients, the ability to connect with family members such as children who may struggle with phone calls, and the empowerment of the healthcare proxy to manage communication. Task success rates and satisfaction scores were high during both rounds of formative usability testing that was conducted with a small, self-motivated population of clinical team members and ICU family members Several usability issues, including difficult navigation, unclear labels, and accessibility concerns, were identified. Difficulties with navigating the app were particularly frustrating for one family user who represents the outlier low SUS score of 17.5 in the second round of usability testing. These issues were addressed through iterative app improvements which included a focus on improving clarity of labels and efforts to make navigation throughout the app easier. Our findings are consistent with prior qualitative work on ICU communication applications, showing that these tools can “open up” communication by making messages clearer and expanding what patients and families are able to express. In that work, family members described feeling relief, less frustration, and less stress when communication became easier (20). Combined, these studies suggest the value of digital communication tools may extend beyond message delivery alone and include meaningful emotional benefits for families.

While overall perceptions of the VoiceLove app were high, there were notable implementation concerns. Many participants raised the concern for VoiceLove to increase nursing workload if patients and their families need help with app troubleshooting. During the observation period, however, some nurses thought that VoiceLove could potentially decrease their workload by cutting down on incoming calls and by decreasing the emotional weight of nurses delivering deeply personal family messages. Some users also noted that learning how to use a technology such as VoiceLove could be burdensome to family members or stressful for family members without access to this technology. These concerns score the need for strong patient and family technologic support to address app issues as they arise. Use of electronic tools such as VoiceLove could also worsen existing social inequities. Families without the ability to use VoiceLove, such as those without smart phones due to financial constraints or those without the necessary literacy to engage with the app, could have less opportunities for engagement and connection with their loved ones. Efforts to implement VoiceLove into existing ICU communication workflows should ensure that this new tool does not take away opportunities for family members to engage with their loved ones through other mechanisms.

Additionally, improved communication may not be universally reassuring. Prior qualitative work found that although families valued enhanced communication, some also experienced sadness or distress when the patient was better able to express fear, worry, or anxiety (20). This suggests that implementation of communication technologies such as VoiceLove should include attention to emotional support, user coaching, equity of implementation, and clear expectations for families during a stressful ICU admission. Voice messages could also be distressing for ICU patients, in particular those who are experiencing delirium or agitation. This is particularly notable as many ICU patients may not be able to initially consent to receiving these messages. ICU patients may lack capacity to consent due to their illness and the healthcare proxy may provide surrogate consent for use of this communication tool. Close attention to nursing workload burden, family psychological impact, impact on family dynamics, and emotional impact on vulnerable ICU patients will need to be emphasized in subsequent studies.

This study is the first step in a larger plan to evaluate the feasibility and efficacy of the VoiceLove app to address family engagement and patient-family communication in the ICU. This formative usability testing was performed to ensure app optimization ahead of a clinical trial where real-world feasibility of VoiceLove can be assessed.

## 5 Limitations

This study does have several limitations. At the start of testing sessions, participants were encouraged to provide open and honest feedback. However, participants' usability ratings may have been influenced due to knowledge that they were being observed or due to desire to please the research team. The usability testing was conducted with a small group of self-motivated participants from a single academic medical center which may limit generalizability of these findings. Some of the positive feedback on the app concept may reflect selection bias given the convenience sample of participants. Given the small sample size, SUS scores must be interpreted with caution noting the variability observed in round two. Finally, the testing was done in a simulated setting which may fail to capture all the usability and implementation concerns that could arise in real-world application. This is particularly true for concerns surrounding nursing workload which cannot be fully captured in a simulated environment.

## 6 Conclusion

VoiceLove is a novel app that may help family members connect to patients in the ICU by sending voice messages without reliance on clinical staff to coordinate communication. Diverse ICU stakeholders viewed the app as a promising way to empower family members and comfort patients who face major barriers to communication. Implementation concerns include nursing workload and burden on families to learn a new technology. This iterative usability testing was used to substantially improve the VoiceLove app, prior to a structured intervention study that will assess the feasibility of its implementation in the ICU.

## Data Availability

The raw data supporting the conclusions of this article will be made available by the authors, without undue reservation.
